# Metaproteome plasticity sheds light on the ecology of the rumen microbiome and its connection to host traits

**DOI:** 10.1038/s41396-022-01295-8

**Published:** 2022-08-16

**Authors:** Goor Sasson, Sarah Moraïs, Fotini Kokou, Kristina Plate, Anke Trautwein-Schult, Elie Jami, Edward A. Bayer, Dörte Becher, Itzhak Mizrahi

**Affiliations:** 1 Faculty of Natural Sciences, Ben-Gurion University of the Negev, Beer-Sheva, 8499000 Israel; 2 Institute of Microbiology, Department of Microbial Proteomics, (Center for Functional Genomics of Microbes), University of Greifswald, Greifswald, 17489 Germany; 3 Department of Ruminant Science, Institute of Animal Sciences, Agricultural Research Organization, Volcani Center, Rishon LeZion, 7505101 Israel; 4 Department of Biomolecular Sciences, The Weizmann Institute of Science, Rehovot, 7610001 Israel; 5 Present Address: Aquaculture and Fisheries Group, Department of Animal Sciences, Wageningen University, 6700AH Wageningen, The Netherlands

**Keywords:** Microbial ecology, Microbiome

## Abstract

The arsenal of genes that microbes express reflect the way in which they sense their environment. We have previously reported that the rumen microbiome composition and its coding capacity are different in animals having distinct feed efficiency states, even when fed an identical diet. Here, we reveal that many microbial populations belonging to the bacteria and archaea domains show divergent proteome production in function of the feed efficiency state. Thus, proteomic data serve as a strong indicator of host feed efficiency state phenotype, overpowering predictions based on genomic and taxonomic information. We highlight protein production of specific phylogenies associated with each of the feed efficiency states. We also find remarkable plasticity of the proteome both in the individual population and at the community level, driven by niche partitioning and competition. These mechanisms result in protein production patterns that exhibit functional redundancy and checkerboard distribution that are tightly linked to the host feed efficiency phenotype. By linking microbial protein production and the ecological mechanisms that act within the microbiome feed efficiency states, our present work reveals a layer of complexity that bears immense importance to the current global challenges of food security and sustainability.

## Introduction

In recent years, the relationship between the bovine host and its rumen microbiome has emerged as a hallmark of host-microbe symbiosis [1]. The rumen is the first compartment of the bovine’s digestive tract and houses a complex microbial community responsible for the fermentation of the ingested feed [2], thus providing up to 80% of the host energy requirements [3]. In order to convert the plant feed to fermentation products, rumen microbes orchestrate a complex fiber degradation and fermentation process that is organized in a trophic-like network [4]. The microorganisms forming these trophic networks maintain a complex community consisting of hundreds of coexisting microbial species from all domains of life with the vast majority belonging to the bacterial domain.

Owing to the immense importance of milk as a nutritional source in the human diet, the bovine host has long been studied with the intent of identifying factors that contribute to animal production. While selective breeding and diet optimization have been the main strategies to improve milk production, studying the rumen microbiome has revealed the link between the rumen microorganisms with host function and production [5–7]. In this context, several studies have emphasized the link between specific microbial species and microbial coding capacity (i.e., metagenomic gene content) with important animal production indices, such as feed efficiency in terms of residual feed intake (RFI), milk yield, methane emission and more [7–12]. These studies have deepened our understanding of the rumen ecosystem by providing estimates regarding the extent to which a given host trait is modulated by ruminal microbiome composition. They further suggested specific microbe-trait links and proposed mechanistic insights into the microbial pathways that distinguish between animals that exhibit different energy harvest feed efficiencies. Nevertheless, in order to better comprehend, predict and potentially modulate the rumen ecosystem, we need to determine how specific microbial populations perceive and interact with the rumen environment. Consequently, we need to deepen our exploration beyond the simple content of microbial genes and delve into their expression characteristics -- i.e., to determine how different microbial species adjust their levels of specific protein production with regard to the microbiome state that they inhabit and to discover how gene expression stands in conjunction with host features, such as feed efficiency.

Previously, we studied the microbial composition, gene content and metabolic output within the rumen of 78 animals and classified them into two host phenotypes with regards to their feed efficiency: efficient and inefficient animals [7]. We found that these two host feed efficiency states were associated with different microbiome states. Specific microbial metabolic pathways and metabolites enriched within feed efficient animals were linked to better energy harvesting and carbon usage characteristics, while reducing methane emission. The two distinct host feed efficiency states arose despite the strictly controlled parameters of the study, with the animals studied belonging to the same breed and from the same herd, reared in the same facility, and fed the same diet. These identical parameters should have eliminated environment-derived variations, further suggesting potential involvement of historical contingency effects and microbial interactions as driving factors [1, 13]. Therefore, our understanding of the actual functions that are implemented by the microbiome members and how individual microbes perceive and interact in these two compositional microbiome states, subjected to the same controlled environmental parameters, is lacking. Indeed, while the study from Shabat et al. [7], sheds important light over the role of individual microbial species and their coding capacities as determinants of feed utilization and methane emission, our capacity to incorporate knowledge concerning the expression level of microbial functions is now imperative.

Here, we investigate whether protein production levels are predictive of host traits and whether microbial taxa differently perceive and interact with their environments in these two community states. Furthermore, we aimed to uncover the potential ecological mechanisms that underlie these community states both in single microbial populations as well as the entire microbial community, by studying microbial proteome production and its relationship to the host feed efficiency state. We therefore selected extreme phenotypes from our previous cohort as subjects in our study [7]. Thus, six highly feed efficient animals and six low feed efficient animals were investigated for their ruminal metaproteomic content, in order to further understand how these ecosystems function and to link microbial activity to their host state. Our results unveiled an unexpected level of plasticity of protein production at the single genotype to the community level that sheds light on both the ecology of these ecosystems as well as the connection to host traits. Finally, our work substantially contributes to the development of currently critically lacking workflows linking metagenomes assembled genomes databases to proteomic data.

## Materials and methods

### Shotgun sequencing and generation of metagenome-assembled genomes

In our previous study, 78 Holstein Friesian dairy cows were sampled for rumen content, metagenomic shotgun sequencing was carried out, and raw Illumina sequencing reads were assembled into contigs using megahit assembler using default settings [7]. We used a pooled assembly of the original 78 samples to increase the quality of the metagenome-assembled genomes (MAGs) with the syntax: *megahit* [14] *-t 60 -m 0.5 −1 [Illumina R1 files] −2 [Illumina R2 files]*. Next, the assembled contigs were indexed using BBMap [15]: *bbmap.sh threads* = *60 ref* = *[contigs filename]*. Thereafter, reads from each sample were mapped to the assembled contigs using BBTools’ *bbwrap.sh* script. In order to determine the depth (coverage) of each contig within each sample, the *gi_summarize_bam_contig_depths* tool was applied with the parameters: gi_summarize_bam_contig_depths --outputDepth depth.txt --pairedContigs paired.txt *.bam --outputDepth depth.txt --pairedContigs paired.txt.

Using the depth information, *metabat2* [16] was executed to bind genes together into reconstructed genomes, with parameters: *metabat2 -t40 -a depth.txt*.

To evaluate genomic bin quality, we used the CheckM [17] tool, with parameters: *checkm lineage_wf [in directory] [out directory] -x faa --genes -t10*.

### Preparing proteomic search library

We generated 93 unique high-quality MAGs, and further increased our MAG database by including phyla that were not represented in our set of MAGs. In order to do so, we used the published compendium of 4,941 rumen metagenome-assembled genomes [18] and dereplicated those MAGs using dRep [19]. We then selected MAGs from phylum Spirochaetes, Actinomycetota, Proteobacteria, Firmicutes, Elusimicrobia, Bacillota, Fibrobacteres and Fusobacteria, which had the highest mean coverage in our samples as calculated using BBMap and *gi_summarize_bam_contig_depths* as described above [15]. This strategy minimized the false discovery rate (FDR), that would have been obtained if larger and unspecific databases would have been employed [20] and allowed the addition of 14 MAGs to our database.

In order to create the proteomic search library, genes were identified along the 107 MAGs using the Prodigal tool [21], with parameters: *prodigal meta* and translated in silico into proteins, using the same tool. Replicates sequences were removed. Protein sequences from the hosting animal (*Bos taurus*) and common contaminant protein sequences (64,701 in total) were added to the proteomic search library in order to avoid erroneous target protein identification originating from the host or common contaminants. Finally, in order to subsequently assess the percentage of false-positive identifications within the proteomic search [22], the proteomic search library sequences were reversed in order and served as a decoy database.

### Proteomic analysis

The bacterial fraction from rumen fluid of the 12 selected animals selected from extreme feed efficiency phenotypes, were obtained at the same time as the samples analyzed for metagenomics and stored at −20 °C until extraction. To extract total proteins, a modified protocol from Deusch and Seifert was used [23]. Briefly, cell pellets were resuspended in 100 µl in 50 mM Tris-HCl (pH 7.5; 0.1 mg/ml chloramphenicol; 1 mM phenylmethylsulfonyl fluoride (PMSF)) and incubated for 10 min at 60 °C and 1200 rpm in a thermo-mixer after addition of 150 µl 20 mM Tris-HCl (pH 7.5; 2% sodium dodecyl sulfate (SDS)). After the addition of 500 µl DNAse buffer (20 mM Tris-HCl pH 7.5; 0.1 mg/ml MgCl2, 1 mM PMSF, 1 μg/ml DNAse I), the cells were lysed by ultra-sonication (amplitude 51–60%; cycle 0.5; 4 × 2 min) on ice, incubated in the thermo-mixer (10 min at 37 °C and 1,200 rpm) and centrifuged at 10,000 × *g* for 10 min at 4 °C. The supernatant was collected and centrifuged again. The proteins in the supernatant were precipitated by adding 20% pre-cooled trichloroacetic acid (TCA; 20% v/v). After centrifugation (12,000 × *g*; 30 min; 4 °C), the protein pellets were washed twice in pre-cooled (−20 °C) acetone (2 × 10 min; 12,000 × *g*; 4 °C) and dried by vacuum centrifugation. The protein pellet was resuspended in 2× SDS sample buffer (4% SDS (w/v); 20% glycerin (w/v); 100 mM Tris-HCl pH 6.8; a pinch of bromophenol blue, 3.6% 2‑mercaptoethanol (v/v)) by 5 min sonication bath and vortexing. Samples were incubated for 5 min at 95 °C and separated by 1D SDS-PAGE (Criterion TG 4-20% Precast Midi Gel, BIO-RAD Laboratories, Inc., USA).

As previously described, after fixation and staining, each gel line was cut into 10 pieces, destained, desiccated, and rehydrated in trypsin [24]. The in-gel digest was performed by incubation overnight at 37 °C. Peptides were eluted with *Aq. dest*. by sonication for 15 min The sample volume was reduced in a vacuum centrifuge.

Before MS analysis, the tryptic peptide mixture was loaded on an Easy-nLC II or Easy-nLC 1000 (Thermo Fisher Scientific, USA) system equipped with an in-house built 20 cm column (inner diameter 100 µm; outer diameter 360 µm) filled with ReproSil-Pur 120 C_18_-AQ reversed-phase material (3 µm particles, Dr. Maisch GmbH, Germany). Peptides were eluted with a nonlinear 156 min gradient from 1 to 99% solvent B (95% acetonitrile (v/v); 0.1% acetic acid (v/v)) in solvent A (0.1% acetic acid (v/v)) with a flow rate of 300 ml/min and injected online into an LTQ Orbitrap Velos or Orbitrap Velos Pro (Thermo Fisher Scientific, USA). Overview scan at a resolution of 30,000 in the Orbitrap in a range of 300-2,000 m/z was followed by 20 MS/MS fragment scans of the 20 most abundant precursor ions. Ions without detected charge state as well as singly charged ions were excluded from MS/MS analysis. Original raw spectra files were converted into the common mzXML format, in order to further process it in downstream analysis. The spectra file from each proteomic run of a given sample was searched against the protein search library, using the Comet [25] search engine with default settings.

The TPP pipeline (Trans Proteomic Pipeline) [26] was used to further process the Comet [25, 27] search results and produce a protein abundance table for each sample. In detail, PeptideProphet [28] was applied to validate peptide assignments, with filtering criteria set to probability of 0.001, accurate mass binning, non-parametric errors model (decoy model) and decoy hits reporting. In addition, iProphet [28, 29] was applied to refine peptide identifications coming from PeptideProphet. Finally, ProteinProphet [28–30] was applied to statistically validate peptide identifications at the protein level. This was carried out using the command: *xinteract -N[my_sample_nick].pep.xml -THREADS* = *40 -p0.001 -l6 -PPM -OAPd -dREVERSE_ -ip [file1].pep.xml [file2].pep.xml.. [fileN].pep.xml* > *xinteract.out 2* > *xinteract.err*. Then, TPP GUI was used in order to produce a protein table from the resulting ProtXML files (extension *ipro.prot.xml*).

Subsequently, proteins that had an identification probability < 0.9 were also removed as well as proteins supported with less than 2 unique peptides (see Supplementary Table 1).

### Quantifying metagenomic presence of MAGs

A reference database containing all 107 MAGs’ contigs was created (*bbmap.sh* command, default settings). Then, the paired-end short reads from each sample (FASTQ files) were mapped into the reference database (bbwrap.sh, default settings), producing alignment (SAM) files, which were converted into BAM format. Subsequently, a contig depth (coverage) table was produced using the command jgi_summarize_bam_contig_depths --outputDepth depth.txt --pairedContigs paired.txt *.bam. As each of the MAGs span on more than one contig, MAG depth in each sample was calculated as contig length weighted by the average depth. Finally, to account for unequal sequencing depth, each MAG depth was normalized to the number of short sequencing reads within the given sample.

### Correlating metagenomic and proteomic structures

In order to compare metagenomic and proteomic structures, we first calculated the mean coding gene abundance and mean production levels of each of the 1629 detected core proteins over all 12 cows. Both mean gene abundance and mean production level were translated into ranks using the R rank function. The produced proteins were ranked in descending order and the coding genes in the gene abundance vector were reordered accordingly. The two reordered ranked vectors then plotted using the R *pheatmap* function, and colored using the same color scale.

### Selection of proteins for downstream analysis

As our goal was to analyze plasticity in microbial protein production in varying environments, e.g., as a function of host state, only MAGs that were identified in all of the 12 proteomic samples were kept for further analysis. Consequently, only proteins that were identified in at least half of the proteomic samples (e.g., in at least six samples) were selected. This last step aimed to reduce spurious correlation results. These filtering steps retained 79 MAGs coding for a total of 1,629 measurable proteins.

### Feed efficiency state prediction and ordination

In order to calculate the accuracy in predicting host feed efficiency state based on the different data layers available (16S rRNA (Supplementary Table 2), metagenomics, metaproteomics), the principal component analysis (PCA) axes for all the samples based on the microbial protein production profiles were calculated. Then, twelve cycles of model building and prediction were made. Each time, the two first PCs of each of five cows along with their phenotype (efficiency state) were used to build a Support Vector Machine (SVM) [R *caret* package] prediction model and one sample was left out. The model was then used to perform subsequent prediction of the left-out animal phenotype (feed efficiency) by feeding the model with that animal’s first two PCs. This leave-one-out methodology was then repeated over all the samples. Finally, the prediction accuracy was determined as the percent of the cases where the correct label was assigned to the left-out sample. For the proteomics data, this procedure was applied on both the raw protein counts, and the protein production normalized based on MAG abundance, which enabled us to compare the prediction accuracies of the microbial protein production to that of the raw protein counts.

### Identification proteins associated with a specific host state

In order to split the proteomics dataset into microbial proteins that tend to be produced differently as a function of the host feed efficiency states, each microbial protein profile was correlated to the sample’s host feed efficiency measure (as calculated by RFI) using the Spearman correlation (R function *cor*), disregarding the *p* value. Proteins that had a positive correlation to RFI were grouped as inefficiency associated proteins. In contrast, proteins that presented a negative correlation to RFI were grouped as efficiency associated proteins. To test for equal sizes of these two protein groups, a binomial test was performed (R function *binom.test*) to examine the probability to get a low number of feed efficient proteins from the overall proteins under examination, when the expected probability was set to 0.5.

### Functional assignment of proteins

Protein functions were assigned based on the KEGG (Kegg Encyclopedia of Genes and Genomes) [31] database. The entire KEGG genes database was compiled into a Diamond [32] search library. Then, the selected microbial proteins were searched against the database using the Diamond search tool. Significant hits (evalue < 5e-5) were further analyzed to identify the corresponding KO (KEGG Ortholog number). Annotations of glycoside hydrolases were performed using dbcan2 [33].

### Protein level checkerboard distribution across the feed efficiency groups

The checkerboard distribution in protein production profiles was estimated separately within the feed efficient and inefficient animal groups. To enable the comparison between the two groups’ checkerboardness level, we chose a standardized C-score estimate (Standardized Effect Size C-score - S.E.S C-Score), based on the comparison of the observed C-score to a null-model distribution derived from simulations. The S.E.S C-score was estimated using the *oecosimu* function from R vegan package with 100,000 simulated null-model communities.

### Calculating functional redundancy

The functional redundancy within a given group of proteins was measured as the mean number of times a given KO occurred within a given group, while neglecting proteins that have not been assigned a KO level functional annotation.

In order to test whether a given group of proteins exhibits more or less functional redundancy than would have been expected, a null distribution for functional redundancy was created, based on the number of proteins in the given group. A random group of proteins was drawn from the entire set, keeping the same sample size as in the tested group, and the process was repeated 100 times. Then, the functional redundancy for each random protein group was calculated. Thereafter, the null distribution was used to obtain a *p* value to measure the likelihood of obtaining such a value under the null.

### Examining functional divergence

Examining the functional divergence between the two groups of proteins, e.g. the feed efficiency and inefficiency associated proteins, was done by first counting the amount of shared functional annotations, in terms of KOs between the two groups. Thereafter, a null distribution for the expected count of KOs was built by randomly splitting in an iterative manner the proteins into groups of the same sizes and calculating the number of shared KOs. A *p* value for the actual count of shared proteins was obtained by ranking the actual count over the null distribution.

### Calculating average nearest neighbor ratio (ANN ratio)

ANN Ratio analysis was carried out independently for each protein function (KO), containing more than 14 proteins with at least 5 proteins within each feed efficiency group. Initially, all proteins assigned to a given KO were split into two sets, in accordance to their feed efficiency affiliation group. Thereafter, proteins within each set were independently projected into two-dimensional space by PCA applied directly to Sequence Matrix [34]. Average nearest neighbor ratio within each set was then calculated within the minimum enclosing rectangle defined by principal component axes PC1 and PC2, as defined by Clark and Evans [35].

### MAG feed efficiency score calculation

Microorganism feed efficiency score was calculated for each MAG individually by first ranking each protein being produced by the given microbe along the 12 animals, based on the normalized protein production levels. Thereafter, a representative production value for the microbe in each animal was calculated as the average of the ranked (normalized) protein production levels in that animal (using R *rank* function). This ranking allowed us to alleviate the potential skewing effect of highly expressed proteins. The microorganism’s Feed Efficiency Score was calculated as the difference between its mean representative production value within feed efficient animals to that within feed inefficient animals. Values close to zero will reflect similar distribution between the two animal groups, positive values will indicate higher expression among efficient animals, and negative values will indicate higher expression among inefficient animals. To calculate significance, the actual feed efficiency score was compared to values in a distribution derived from a permutation based null model. Each of the permuted Feed Efficiency Scores (10,000 for each microbe) was obtained by independently shuffling each of the proteins produced by the MAG between the animals, prior to calculating the actual microorganism feed efficiency score. By positioning the absolute score value over its distribution under permuted assumptions (absolute values), we obtained a significance *p* value.

### MAG phylogenetic tree construction and phylogenetic signal estimation

In order to assess the link between phylogenetic similarity between the MAGs and their association with feed efficiency, phylogenetic tree estimating evolutionary relationships between the MAGs was constructed using the PhyloPhlAn pipeline [36]. The phylogenetic signal for Microorganism Feed Efficiency Score was estimated by providing the phylogSignal function from R phylosignal [37] package with MAGs phylogenetic tree and respective values. Pagel’s Lambda statistics was chosen for the analysis, owing to its robustness [38].

### Plot generation

All bar plots, scatter plots and other point plots were generated with R package ggplot2. Heatmaps were produced by either ggplot2 [39] or pheatmap [https://cran.r-project.org/web/packages/pheatmap/index.html] R packages. KEGG map was produced using the online KEGG Mapper tool [40]. Phylocorrelogram was produced with phyloCorrelogram function from R package phylosignal [37].

### MAG differential production analysis

MAGs that contain a minimal number of proteins (50 functions) were selected for differential protein production analysis, in order to have sufficient data to perform statistical tests. For each MAG, the relative production was used in order to calculate the Jaccard pairwise dissimilarity for core protein production between feed efficient and inefficient cows using the R vegan package. Analysis of similarity between efficiency and inefficiency associated proteins for each MAG (ANOSIM) values and *p* values were then calculated using the same package.

### Predicting animal feed efficiency state according to GH family counts

Using all GH annotated proteins, a feature table that sums the count of each GH family within each sample was produced. Thereafter a leave-one-out cross-validation (LOOCV) [R *caret* package] was performed, each time building a Random Forest (RF) prediction model from the GH family counts and efficiency state of 11 samples, leaving one sample outside. Each one of the RF models, in its turn, was applied on the left-out animal to predict its efficiency state. Model accuracy and AUC curve were calculated based on the LOOCV performance.

## Results

### Proteome structure is incongruent with metagenome structure and is more predictive of host traits

To delineate the link between the rumen microbial composition, feed efficiency phenotype and other host traits, we examined whether and how the protein production patterns of the rumen microbiome can explain host traits via metaproteomic analysis of a cohort of 12 animals on the most extreme end of the feed efficiency phenotypes, with 6 animals exhibiting the highest feed efficiency and 6 animals exhibiting the lowest feed efficiency. Protein search and identification was performed using assembled metagenomic assembled genomes (MAGs) from metagenomic reads that originated from these samples as a reference database. Our metagenomic assembly effort resulted in 93 unique high-quality MAGs, mostly affiliated with the Firmicutes and Bacteroidetes phyla, as well as Actinobacteria, Tenericutes, Proteobacteria, Elusimicrobia and Euryarchaeota (Supplementary Fig. 1), these MAGs were augmented with 14 additional MAGs from a rumen genome collection [18] selected to expand the phylogenetic diversity and better represent the typical rumen metagenomic content as described before [38] [https://cran.r-project.org/web/packages/pheatmap/index.html]. Using the predicted open reading frames (ORFs) of these MAGs we identified a total of 5574 proteins in our samples, of which 1629 were defined “core” proteins (Supplementary Table 3). We defined proteins as “core” proteins if they were present in at least six of the twelve animals and are expressed by microbes that were metagenomically identified in all samples.

The proteomic data corroborated patterns highlighted in our previous study [7]. We have previously reported that there is a higher short chain fatty acid production in the efficient animals [7], here we could also detect higher production of proteins involved in propionate and acetate productions in feed efficient animals (Supplementary Fig. 2).

**Fig. 1:**
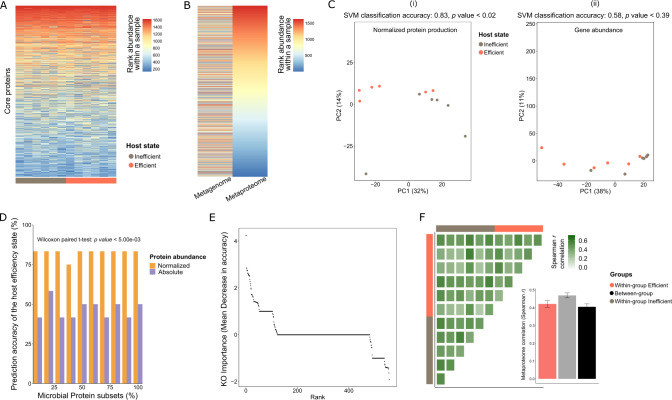
Normalized proteomics is an accurate and sensitive predictor for feed efficiency states. **A** Heatmap showing the highly conserved structure of the rumen metaproteome across the 12 individual animals. Columns represent the samples, and rows represent the 1629 identified core proteins. **B** Comparative ranking heatmaps of genes normalized by abundance from the metagenome (left) and their corresponding protein from the metaproteome (right). Color gradient in **A**, **B** indicates the rank abundance of a given gene or protein within a given sample. **C** Ordination plots using normalized core protein production (i, left) or their corresponding coded gene abundance (ii, right) of the 12 individual animals, with the SVM classification accuracy and *p* value for each one of the datasets shown above the plots. **D** Bar-plot depicting the prediction accuracy of the host feed efficiency state based on the core protein production. The *X*-axis represents the percentage of randomly selected microbial protein subsets based on which the Support Vector Machine (SVM) model was built. The y-axis represents the prediction accuracy for the animal feed efficiency state in %. Analysis was performed on both the normalized (orange) or absolute (purple) protein abundance. **E** Rank-plot showing the varying importance of different microbial functions (KOs) for the prediction of host feed efficiency. The *x*-axis represents the KOs ranked by decreasing importance based on Random Forest Mean Decrease in Accuracy measure, as indicated by the *y*-axis. **F** Heatmap showing the protein production levels between and within host feed efficiency state groups estimated by Spearman *r* correlation. Feed inefficient animals exhibited greater hierarchical structure (mean *r* = 0.52) than feed efficient animals (mean *r* = 0.45) after *t*-test (*p* value < 0.02), as summarized in the inset bar-plot.

The overall structure of the identified core proteome, as determined by the individual protein production levels, was found to be highly conserved across the 12 samples, regardless of their feed efficiency phenotypes (Fig. 1A). Interestingly, the observed recurring structure of the proteome did not correspond to the structure of the metagenome, where the protein production levels of individual proteins did not match their corresponding gene abundance (Fig. 1B). Following this observation, we asked whether proteome structure more strongly reflects ecosystem function rather than its gene content and could serve as a better predictor to distinguish host phenotype.

### Proteomics overpowers metagenomics and taxonomic composition as an accurate predictor for feed efficiency state

To assess whether protein production can serve as a predictor for animal feed efficiency states, we analyzed and compared its discriminating potential, with respect to those of our additional datasets (e.g., 16S rRNA amplicon sequencing, metagenomes). For the proteome dataset, we applied either raw (absolute) protein production abundances or protein abundance normalized by MAG abundance, the latter, termed normalized protein production. This normalization procedure was performed in order to dissociate between the activity of a given bacterial population to its abundance in the ecosystem (Fig. 1C). The results of our analysis show that the most discriminating data layer for the two host feed efficiency states is the normalized protein production (Fig. 1C (i), accuracy = 0.83 and *p* = 0.02). We performed the same analysis using 16 S rRNA amplicon sequencing, genome abundance of the examined MAGs, ORF abundance of the 5574 proteins that were shown to be produced in the metaproteomic analysis (shotgun metagenome) and abundance of 5574 produced proteins (metaproteome). These analyses revealed an overall lower discriminatory power for these datasets compared to the normalized proteome originating from the examined MAGs but with superior accuracy for the metaproteome and the 16S rRNA amplicon sequencing datasets (SVM/LOO 16S rRNA amplicon, *p* = 0.07, accuracy = 0.75 Supplementary Fig. 3A; MAGs genomes abundance, *p* = 0.61, accuracy = 0.5 Fig. 1C (ii); metaproteome abundance with total protein production *p* = 0.07 accuracy = 0.75, Supplementary Fig. 3B; and shotgun metagenome abundance, *p* = 0.61 accuracy = 0.5, Supplementary Fig. 3C). The non-normalized protein production data also encompassed a less accurate discriminator of the two host feed efficiency phenotypes as determined by support vector machine classification with a leave-one-out (SVM/LOO) approach (Supplementary Fig. 3D and Fig. 1D), we find that feed efficiency state prediction clearly exhibited higher accuracy, based on the normalized protein counts compared to absolute counts where the prediction accuracy dropped to about 50%, which constitutes random chance. This emphasizes the importance of the individual population function decoupled from its abundance to accurately predict host feed efficiency state based on protein production (Supplementary Fig. 3B and Fig. 1D). To increase the resolution of prediction and to measure the extent by which the metaproteome production profile predicts the feed efficiency state with a minimal set of proteins, we randomly sampled 10 to 100% of the 1629 produced core proteins to predict host phenotype. Our results revealed that even a randomly selected subset of 10% of the proteins identified can be used to predict the host feed efficiency state with an accuracy of up to 80% (the test was performed ten times, each time a subset of 162 proteins was randomly selected) (Fig. 1D). To further determine if indeed 10% of the proteins are representative of the overall variance attributed to the examined MAGs, we examined how well the 10% subset of the proteins (10%) correlates with the remaining 90% by creating two Bray–Curtis similarity matrices, one based on 10% of the core proteins and the other based on the 90% of core proteins, and performed a Mantel test between them. We obtained a substantial correlation (*r* = 0.856 as opposed to *r* = 0.004 for the null model, *p* < 0.001). These results suggest that a small proportion of the proteome is sufficient to represent most of the variance of the overall proteome and could be due to coexpression of the majority of the proteins in each of the microbiome states therefore creating a statistical linkage.

We next used a decision tree-based approach for determining the importance of produced KEGG Orthology groups (KOs) for their ability to discriminate between the feed efficiency phenotypes. For this purpose, we used the random forest algorithm to rank all the normalized produced KOs for their importance in predicting the association of a given animal to the two groups (Fig. 1E). Interestingly, our analysis shows from the top 10 most discriminating functions revealed by random forest, 7 belong to the KEGG “metabolism” level, and include specifically carbohydrate metabolism of polysaccharide and simple sugar utilization and fermentation (Supplementary Fig. 4). These findings indicate that despite the conserved structure of the proteome across cows, differences in protein production led to a strong discriminatory power of the normalized proteome of the examined MAGs to predict the feed efficiency state of the animal. Moreover, these findings also reveal that individual microbial populations have specific and consistent protein production profiles unique for each of the two feed efficiency states. Indeed, when we assessed the similarity in rumen proteome structure across animals, we found that microbiomes belonging to the same feed efficiency group exhibit a significantly higher similarity and correlation to one another, when compared to similarity and correlation between the groups (Fig. 1F, F(i)). Furthermore, the portion of KOs shared between the feed efficiency and inefficiency associated proteins was significantly lower than expected by chance (Supplementary Fig. 5). In addition, the low feed efficiency animal group exhibited a higher correlation and similarity among the animals, compared to the high feed efficiency group (Fig. 1F). These overall findings directed our attention towards exploring potential ecological mechanisms that could play a role in producing such patterns.

### Evidence of lower redundancy and higher niche partitioning in feed efficient cows

**Fig. 2:**
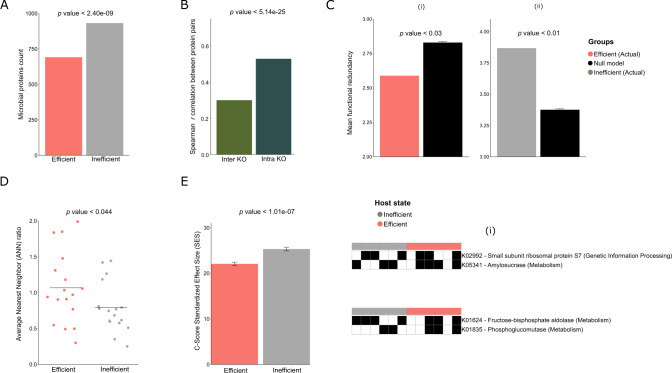
Lower functional redundancy and higher niche partitioning in feed efficient cows. **A** Bar-plot comparing the number of microbial proteins associated with feed efficient animals or inefficient animals. Significance was evaluated with a binomial test. **B** Bar-plot presenting Spearman correlation between pairs of normalized protein production profiles of core proteins, producing the same (intra) or different KO functions (inter). The analysis was performed on the 1629 core proteins without distinction of host feed efficiency state. Significance was tested with *t*-test. **C** Bar plots for functional redundancy calculated as the number of times unique KOs appear in (i) feed efficient and (ii) inefficient protein groups, compared to a random null model. The null model was calculated by randomly splitting the core proteome into subsets of the same sizes as the two actual groups and calculating the mean redundancy (1000 iterations) for each. **D** Average nearest neighbor (ANN) ratio analysis revealed a higher clustering tendency within the feed inefficient animals (ANN < 1, indicating more clustering) compared to feed inefficient (ANN > 1, indicating more dispersion). **E** Bar-plot showing that within feed inefficient animals, proteins are more often co-excluding each other compared to those of feed efficient animals, as calculated by Standardized Checkerboard Index analysis (SES C-score). For example, (i) two proteins coding for two different KOs: phosphoenolypyruvate carboxykinase and enoyl-CoA hydratase, exhibited co-exclusion in feed inefficient animals and co-presence in feed efficient animals. Black indicates the presence and white the absence of a protein from an animal sample.

In our previous study, we demonstrated that feed inefficient animals are characterized by higher microbial diversity both in terms of taxa and coding capacity compared to feed inefficient animals [7]. We therefore asked whether or not this pattern remains at the protein production level. By correlating animal feed efficiency as RFI values to each individual protein, we indeed identified that more proteins are associated with feed inefficiency in animals (i.e., positively correlated with RFI; n = 932) compared to those associated with the feed efficient phenotype (negatively correlated to RFI; *n* = 691). This distribution of proteins associated with each phenotype significantly deviates from the null expectation (*p* < 2.40e − 09, binomial test) (Fig. 2A). Following this finding, we explored our data for a potential functional or ecological mechanism which may explain this recurrent observation across the different data layers. To this end, we analyzed the protein production patterns of the different produced proteins within the context of their functions. We found that proteins coding for the same function exhibited a significantly higher correlation between their normalized counts, compared to proteins that code for different functions (*r* = 0.528, *p* value < *p* < 5.1439e − 25 compared to *r* = 0.3) (Fig. 2B). Hence, the data suggest that protein production is likely driven by environmental conditions, thereby dictating similar functions to be expressed across individual microorganisms.

Following this notion, we asked whether the observed higher richness of produced proteins in the feed inefficient microbiomes is associated with a higher number of different or similar functions which would therefore reflect either increased functional richness or increased functional redundancy. To delineate these two scenarios, we counted the number of produced proteins that are assigned to identical orthologous groups (functionally redundant) versus those that belong to different orthologous groups (functionally different) within each of the feed efficient animal phenotypes. We observed significantly higher counts of feed inefficiency associated proteins than feed efficiency associated ones (Supplementary Fig. 6A). Furthermore, even if both feed efficient and inefficient animals contained proteins involved with carbon and additional metabolism, feed inefficient animals almost systematically exhibited higher counts for each of the functional categories (Supplementary Fig. 6A).

In addition, we find that proteins that were classified as feed inefficiency associated exhibited a higher functional redundancy, i.e., ORFs that code for the same function co-occur significantly more than expected by random chance in the same sample (*p* value < 0.03, Fig. 2Ci). In contrast, functional redundancy of proteins classified as feed efficiency associated had a lower than expected co-occurrence pattern, suggesting a higher functional richness in feed efficient proteomes (*p* < 0.01, Fig. 2Cii). When we measured the phylogenetic distance of proteins assigned to the same orthologous protein groups, we found that they exhibited a higher phylogenetic distance in the high feed efficiency associated proteins (Wilcoxon paired rank-sum test *p* < 0.044, Fig. 2D). Together with our results of lower functional redundancy in the high feed efficiency associated proteins, the finding of orthologous proteins that exhibit a higher phylogenetic distance could potentially suggest a higher degree of niche partitioning at the enzyme level in the feed efficient microbiome state that might reduce competition. We therefore used the Checkerboard score (C-score) [41] as an indicator of competition at the protein level. Indeed, we found that proteins in the feed efficient microbiome states exhibit a significantly lower C-score compared to those from feed inefficient states (Fig. 2E), further indicating lower competition at the protein level. Thus, our results show that inefficient cows’ proteome is characterized by a higher redundancy compared to efficient cows while efficient cows are characterized by a higher functional diversity and lower competition at the protein level.

### Protein production profile diverges on the taxonomic and functional level as a function of feed efficiency state

**Fig. 3:**
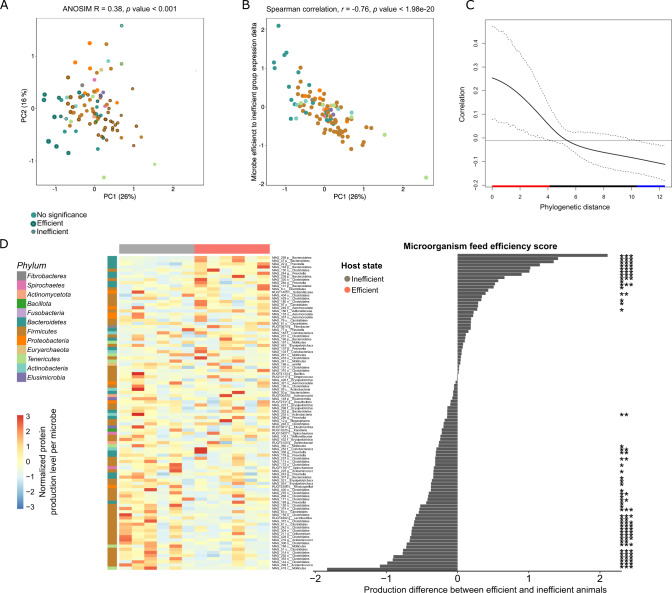
Protein production in metagenome-assembled genomes is shaped by phylogeny. **A** Principal component analysis (PCA) displaying individual total protein production profile of the 100 metagenome-assembled Genomes (MAGs) in the twelve animals. Color represents the MAG taxonomy, while radius size correlates with the microorganism feed efficiency score. Microorganism feed efficiency score is calculated as the mean difference in rank protein production between feed efficient and inefficient animals. **B** Scatter plot displaying the ordination of MAG protein production profiles (*X*-axis) and the microorganism feed efficiency scores (*Y*-axis). Correlation is shown between MAG protein production profile ordination on PC1 that is the highest in explaining production variance (26%) and microorganism feed efficiency score (Spearman correlation coefficient indicated). For each microbe, the production profile was obtained by averaging all of its protein production after they were scaled. **C** Microorganism feed efficiency scores are correlated to their phylogenetic background, with a significant phylogenetic signal, Lambda = 0.2498, *p* value < 0.001. A graph is shown representing the auto-correlation between the microorganism feed efficiency score and phylogenetic distance (*y* axis) over increasing phylogenetic distance. Red, black and blue stand for significant positive, insignificant or significant negative correlation, respectively. **D** Heatmap displaying the protein production levels of 100 core MAGs (left) and their microorganism feed efficiency scores (right). Significance, as shown by asterisks on the right, was obtained by a 10,000 iterations wide null-model distribution, in which the feed efficiency score was repeatedly calculated after randomly and independently shuffling each protein production levels within the samples. */**/*** denote *p* values lower than 0.5/0.005/0.0005, respectively.

Are the different ecological mechanisms acting on each of the feed efficient microbiome states reflected in the protein production profile of single genomes and their specific phylogenies? To address this question, we analyzed the individual protein production profile of each MAG as an input for principal component analysis (PCA). Our analysis indicated that individual MAG protein production profiles are significantly clustered according to their phylum, thus suggesting that rumen microbial taxonomy and expressed functionality are interconnected (ANOSIM 0.38; *p* < 0.001; Fig. 3A). We next asked to what extent the variance of MAG protein production along the microbial expression PCA (PC1, 26% of the variation) correlates to the microbes’ feed efficiency score (Fig. 3B). This analysis revealed a high and significant correlation between the feed efficiency state and the protein production profile of the different MAGs, which was also associated with their phylogeny. We found that the protein production profile of MAGs associated with the *Firmicutes* phylum corresponds to lower feed efficiency states, while the protein production profile of MAGs belonging to the Bacteroidetes, Actinobacteria and Proteobacteria phyla is associated with high feed efficiency (Fig. 3B, Supplementary Fig. 6B). Hence, MAGs which are more closely phylogenetically related exhibit the same association with the feed efficiency state. More globally, when we examined all pairwise phylogenetic distances between the MAGs (Fig. 3C), we found that phylogenetically closer species typically associate with the same feed efficiency state - i.e., the more phylogenetically similar the species, the more likely they are to associate with the same feed efficiency phenotype, as indicated by a substantial and significant phylogenetic signal, Lambda = 0.2498 (*p* < 0.0001; Fig. 3C). Interestingly, when we examine the protein production profile from the single genome level, we find that MAGs have a significant tendency to produce proteins associated with a given feed efficiency state (Fig. 3D and Supplementary Fig. 6B). This finding was obtained by counting the proteins produced from a given genome that are either positively or negatively correlated to feed efficiency and whether we could identify a tendency per genome to produce proteins that are associated with one state over the other compared to random distribution (Fig. 3D and Supplementary Fig. 6B). We also observed that the actual distribution of the proteins that are produced from a given genome significantly deviates from randomness (permutations *p* value < 0.001, Supplementary Fig. 7).

### Microbial activity and plasticity at the single genome level underlie the host feed efficiency state

**Fig. 4:**
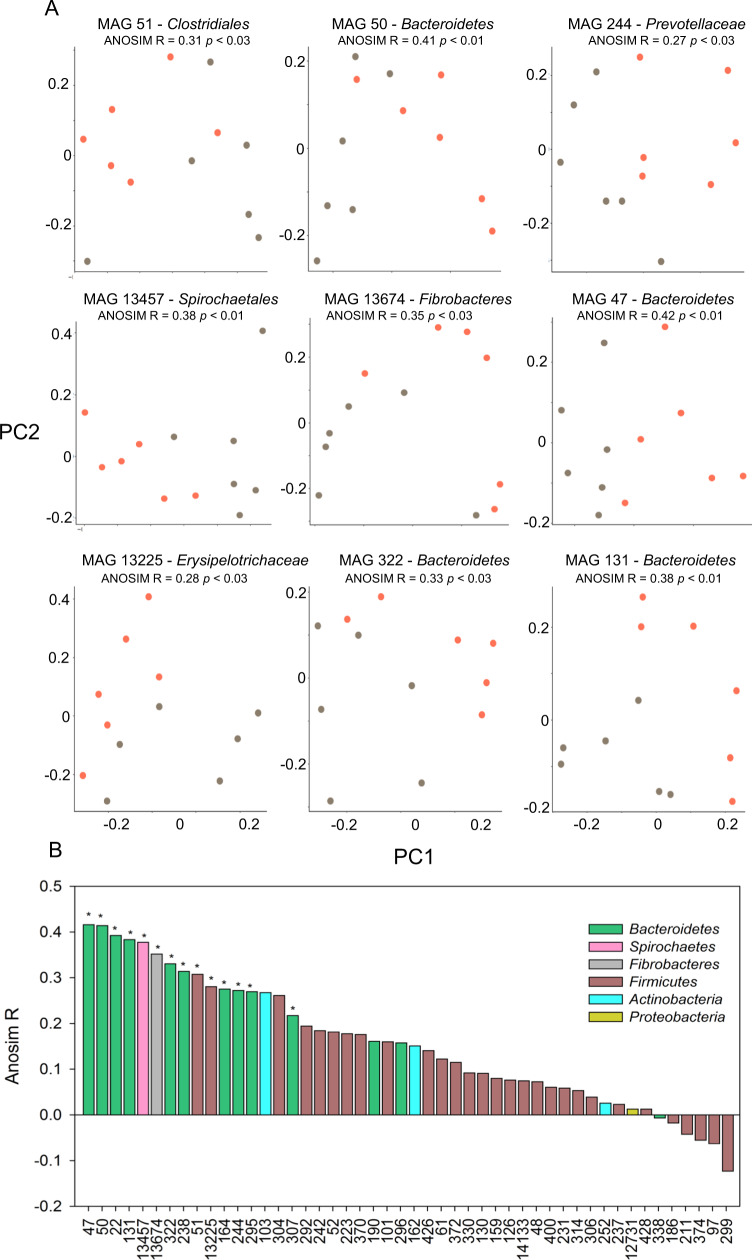
Microbial activity and plasticity at the single genome level. **A** Jaccard dissimilarity of selected MAGs producing different genes across the different feed efficiency states. ANOSIM R values and *p* values for each MAG are shown above each plot. **B** ANOSIM R values for all the MAGS present in all the cows for which at least 40 proteins were identified. The asterisk above each bar denotes the significance for the ANOSIM test for a specific MAG (*p* ≤ 0.05).

Our findings raise the question of whether differential protein production within the same MAG can be observed as a function of the two feed efficiency states. Indeed, when we examined the protein production profile at the single MAG level, we found that single MAGs show differential production in the two states (Figs. 3D and 4A), despite the fact that the MAGs exist in all animals that are fed the same diet. This phenomenon is reflected in the total level of proteome production from a single MAG based on the normalized absolute abundance of proteins produced in each MAG (Fig. 3D) and in the different identity of the produced proteins as exhibited by the PCoA based on the Jaccard presence absence dissimilarity metric of the produced proteome from single MAGs (Fig. 4A, B). In the latter analysis, 15 out of 48 MAGs for which at least 50 functions could be identified, and that are present in all animals showed a significant clustering of the produced proteome as a function of the feed efficiency state (ANOSIM *p* ≤ 0.05; Fig. 4A, B). Permutation test, between the ANOSIM R values obtained and random permutations on each MAGs show that the average values deviate from random (Supplementary Fig. 8).

**Fig. 5:**
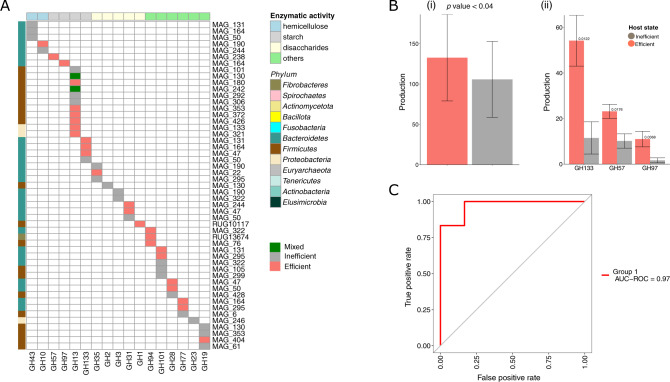
Fiber-degrading enzymes are markers for animal feed efficiency state. **A** Plasticity of the fiber-degrading enzymes production at the single genome level. Presence–absence heatmap of the produced GHs by the different MAGs in feed inefficient, efficient or both host states. Only MAGs producing GHs are represented. The rows represent a given MAG (number and phylogenies are indicated). Columns heatmap represent specific GH families grouped per MAG. **B** Differential absolute production of total GHs (i) or specific starch-related GHs (ii) between efficient and inefficient animals, *p* values from permutation *t*-tests are indicated. **C** Prediction of host efficiency state based on absolute counts of the different GH production. Model accuracy is represented in red.

This differential protein production can be also seen in the functions associated with the different feed efficiency states. Using KEGG and CAZy annotations, we observed that the feed efficiency differential protein production from single MAGs is distributed across various functional categories, including carbohydrate-degrading functions, which are central for rumen metabolism (Fig. 5A). We found that the protein production profile of the glycoside hydrolases (GH) from a given MAG present in all the cows exhibited specificity to one of the feed efficiency states, ie, a specific GHs is either associated with the feed efficient state or the feed inefficient state but not in both (Fig. 5A). This analysis also revealed an interesting observation of specificity of some of the GH functions to a feed efficiency state. For example, amylases GH57 and GH97 were specifically associated with the feed efficient state together with putative cellulases or related enzymes (e.g., GH6 and GH94; Fig. 4B). Interestingly, this analysis of individual microbial populations also highlighted a niche partitioning pattern among the different MAGs at the single enzyme level. Such niche partitioning is apparent when analyzing the production of the total KOs or GHs (Fig. 5A and Supplementary Fig. 9). A total of 120 functions out of 269 (45%) were associated with both feed efficiency states but never from the same MAG, despite the MAG occurrence in the two microbiome states (Supplementary Fig. 9). This phenomenon is very apparent for GH13, a GH family that is present in many MAGs (see Fig. 5A), both in feed efficiency- and inefficiency-correlated proteins but is highly associated with different MAGs between the two feed efficiency groups. This GH family includes starch- and pullulan-degrading enzymes, i.e., polysaccharides encompassing the largest proportion of the animal concentrate feed. For this GH family, MAGs 126, 180, 353, 372, 426, 133 and 321 produced GH13 proteins in the feed efficient state, while MAGs 101, 292 and 306, produced GH13 proteins in the feed inefficient state only. Similarly, GH77 and GH133, were also associated with both host states, but produced from different MAGs (Fig. 5A). This differential production phenomenon was also apparent for GH101 proteins (lysozyme enzymes) which were produced by different MAGs that are present in both feed efficiency states, such as MAG 131 and 295 that produced them in the feed efficient state, while in the feed inefficient state they were produced by MAGs 322, 105 and 299. In addition, we observed more MAGs producing more diverse GH families and specific types of starch-degrading enzymes in the feed efficient animals than in the feed inefficient animals (Fig. 5B). Moreover, the raw abundances of GHs detected in the samples serve a remarkable predictor of the animal feed efficiency state with a calculated accuracy of 0,916 (*p* value < 1.13e − 7) (Fig. 5C). Finally, the protein production profile from the MAGs exhibited a taxonomic tendency, where, in most cases, Firmicutes-associated MAGs produced more feed inefficiency associated GHs, and Bacteroidetes MAGs produced more feed efficiency associated GHs (Fig. 5A).

## Discussion

Our previous work and that of others have established that specific host traits related to production feed efficiency in ruminants can be strongly linked to the structure and gene content of the microbial community of the rumen [1, 7, 8, 42]. However, differences between genomic potential, actual microbial activity and protein production are subject to a myriad of factors, which may result in vast discrepancies between what is seen within the microbes’ genomes and what is produced in a given host or microbiome state [10, 43]. Depending on local abiotic factors, such as individual animal’s physiology and biotic interactions between microbes, an individual microbe may sense vastly different environmental conditions, which could affect its protein production patterns [43–45].

In the current work, we optimize the procedure for extraction and identification of rumen proteins that, when compared to previous studies, allowed us to substantially improve the number of proteins identified [23, 46–48]. We investigated the rumen microbiome state through its produced proteome profile and its link to the host feed efficiency phenotype, previously characterized as containing different coding potential [1, 7, 8, 42]. We asked whether different rumen ecosystems are supported by a divergent microbial physiology, as reflected by their microbial gene production, and whether the latter more accurately reflects host phenotypic state, compared to microbial taxa and gene composition. These questions arose from the initially observed clear dissociation between proteome rank profiles compared to metagenome rank abundance of genes in this study, Indeed the proteome profile of examined prokaryotic rumen MAGs was shown to be significantly more predictive of the feed efficiency phenotype of the cows sampled than any other data layer suggesting that the protein level carries more information relating to the feed efficiency state of the host and ecosystem function. These conclusions that refer only to the examined MAGs, represent specific markers for determining the feed efficiency state. Furthermore, the protein production pattern associated with each of the microbiome states enabled us to accurately predict the host feed efficiency state and also pinpointed specific discriminating functions, encompassing several genes related to carbon and fermentation metabolism, that could be further used as microbiome biomarkers for improving sustainability of husbandry protocols by selecting for more feed efficient microbiomes. These results highlight the importance of multi-level omics analyses, capable of discerning such genome-level nuances in protein production, in light of host phenotype. Moreover, these results led to the hypothesis that ecological forces may enforce different gene expression patterns at the single microbial population level between these two states that will ultimately reflect its environmentally driven realized niche.

Our results indeed revealed that, beyond the globally different protein production patterns found across different animals with contrasting feed efficiency phenotypes, an unexpected plasticity in protein production exists at the single genome level. Strikingly, the same individual MAG found in all of the animals exhibited different protein production between the two feed efficiency states, as well as significantly different overall levels of protein production. Additionally, we find that across the MAGs, the host state is demarcated by different phylogenies, with Bacteroidetes genomes exhibiting increased protein production in the efficient state, whereas Firmicutes genomes are more active in feed inefficient cows (Fig. 3).

These findings suggest that microbial populations at the two microbiome states are subjected to different external stimuli, leading a given microbe to produce different genes. Such stimuli can either be the result of abiotic factors such as intrinsic features of the host that might partly be connected to its genome (e.g., pH regulation via saliva production, retention time via rumen size) or may be due to biotic interactions between the rumen microbiome members. Our results strongly suggest that in each of the microbiome states, the additional biotic effects of the general microbial populations in the surroundings may play a significant part in defining the protein production patterns of the individual population. Hence, although the environmental conditions such as diet and husbandry regimes are similar, the microbial environment that the same microbial populations are experiencing in the two efficiency states is different as reflected by the differential expression of their proteomes. We also identify evidence for niche partitioning at the protein production level, where a given enzyme such as a fiber-degrading enzyme will be produced only from one population and not the other, despite the potential of both to do so in a given host state. Moreover, it seems that different ecological mechanisms play a role in defining specific gene arrays that are produced in each feed efficiency state. In a feed efficient microbiome state, microbes exhibited a significantly lower functional redundancy in protein production compared to the feed inefficient state. The latter state also exhibited a high C-score, suggesting an increased level of competition. Furthermore, even when microbial genes from a feed efficient animal are assigned to the same putative function, they exhibit a larger phylogenetic distance, suggesting more divergent functions and enzyme specialization [49], as was shown for diversification of orthologous microbial enzymes [50]. Overall, these results suggest that niche partitioning at the enzyme level leads to divergent specialized functions and is connected to the feed efficiency phenotype. Recently, niche compartmentalization was also suggested by Hagen et al. [20], while studying cow rumen metaproteome. Indeed, the authors observed that the fungal population produces distinct and complementary fiber-degrading enzymes, and cellulose-degrading genes in particular, than the bacterial populations. Functional redundancy has often been suggested to represent a mechanism of ecosystem stability [51]. Here, we propose that it could also lead to less feed efficient microbial communities (in the context of agriculture as opposed to ecological productivity, which refers to the rate of generation of biomass in an ecosystem). An explanation for this observation could be that lower functional redundancy reflects a more coordinated inter-microbial interaction. With less competition over resources, more energy could instead be utilized into higher yield of final fermentation products, as was measured at the feed efficient state in our previous work [7]. Such products, as important short chain fatty acids like propionate and acetate, then affect, in their availability, the growth and yield of the host animal. The increase in stability, concomitant with functional redundancy, may also explain the significantly higher correlation in protein production in feed inefficient cows compared to feed efficient cows, thus suggesting that feed inefficiency is characterized by a narrower functional state, while feed efficiency may be accompanied by differing functional states [52, 53].

This study strengthens our current understanding of the ecological forces that are in play within the rumen microbiome and their connection to host feed efficiency. As hypothesized, protein production pattern is a more robust indicator of animal phenotype when compared to taxonomic composition and coding potential, i.e., the metagenome. More surprisingly, the high predictive ability of the proteome to categorize animals based on the main phenotype studied here, i.e., feed efficiency is based on global features of protein production, which means that accurate categorization can be made using only a defined subset of proteins. We also propose microbial functional redundancy and competition as drivers of broad phenotypic variation between hosts, which may be of relevance for any ecosystem, not necessarily limited to host-associated ones. These results can potentially be leveraged for future design of synthetic microbial communities. Future studies should focus on the mapping of inter-microbial behavioral interactions, along with experimental validations, in order to integrate and exhaust current omic technologies and control and improve sustainable agriculture.

## Supplementary information

Supplementary Figures and Legends

Supplementary Table 1

Supplementary Table 2

Supplementary Table 3

## Data Availability

Assembled MAGs were deposited in the figshare server https://figshare.com/s/2a1edeb1c1a194933a27. The mass spectrometry proteomics data have been deposited to the ProteomeXchange Consortium via the PRIDE [54] partner repository with the dataset identifier PXD033418.

## References

[1] Mizrahi I, Wallace RJ, Moraïs S (2021). The rumen microbiome: balancing food security and environmental impacts. Nat Rev Microbiol.

[2] Mizrahi I. Rumen symbioses. In: Rosenberg E, DeLong EF, Lory S, Stackebrandt E, Thompson F, editors. The prokaryotes: Prokaryotic Biology and Symbiotic Associations. Berlin, Heidelberg: Springer Berlin Heidelberg; 2013:533–44.

[3] Bergman EN (1990). Energy contributions of volatile fatty acids from the gastrointestinal tract in various species. Physiological Rev.

[4] Morais S, Mizrahi I (2019). Islands in the stream: From individual to communal fiber degradation in the rumen ecosystem. FEMS Rev Microbiol.

[5] Jami E, White BA, Mizrahi I (2014). Potential role of the bovine rumen microbiome in modulating milk composition and feed efficiency. PLoS ONE.

[6] Roehe R, Dewhurst RJ, Duthie C-A, Rooke JA, McKain N, Ross DW (2016). Bovine host genetic variation influences rumen microbial methane production with best selection criterion for low methane emitting and efficiently feed converting hosts based on metagenomic gene abundance. PLOS Genet.

[7] Shabat SKB, Sasson G, Doron-Faigenboim A, Durman T, Yaacoby S, Berg Miller ME (2016). Specific microbiome-dependent mechanisms underlie the energy harvest efficiency of ruminants. ISME J.

[8] Wallace RJ, Sasson G, Garnsworthy PC, Tapio I, Gregson E, Bani P (2019). A heritable subset of the core rumen microbiome dictates dairy cow productivity and emissions. Sci Adv.

[9] Li F, Li C, Chen Y, Liu J, Zhang C, Irving B (2019). Host genetics influence the rumen microbiota and heritable rumen microbial features associate with feed efficiency in cattle. Microbiome.

[10] Kamke J, Kittelmann S, Soni P, Li Y, Tavendale M, Ganesh S (2016). Rumen metagenome and metatranscriptome analyses of low methane yield sheep reveals a Sharpea-enriched microbiome characterised by lactic acid formation and utilisation. Microbiome.

[11] Huws SA, Creevey CJ, Oyama LB, Mizrahi I, Denman SE, Popova M (2018). Addressing global ruminant agricultural challenges through understanding the rumen microbiome: past, present, and future. Front Microbiol.

[12] Cunha CS, Veloso CM, Marcondes MI, Mantovani HC, Tomich TR, Pereira LGR (2017). Assessing the impact of rumen microbial communities on methane emissions and production traits in Holstein cows in a tropical climate. Syst Appl Microbiol.

[13] Moraïs S, Mizrahi I (2019). The road not taken: the rumen microbiome, functional groups, and community states. Trends Microbiol.

[14] Li D, Luo R, Liu C-M, Leung C-M, Ting H-F, Sadakane K (2016). MEGAHIT v1.0: A fast and scalable metagenome assembler driven by advanced methodologies and community practices. Methods.

[15] Bushnell B. BBMap: a fast, accurate, splice-aware aligner. Berkeley, CA, USA: Lawrence Berkeley National Lab (LBNL); 2014.

[16] Kang DD, Li F, Kirton E, Thomas A, Egan R, An H (2019). MetaBAT 2: an adaptive binning algorithm for robust and efficient genome reconstruction from metagenome assemblies. PeerJ.

[17] Parks DH, Imelfort M, Skennerton CT, Hugenholtz P, Tyson GW (2015). CheckM: assessing the quality of microbial genomes recovered from isolates, single cells, and metagenomes. Genome Res.

[18] Stewart RD, Auffret MD, Warr A, Walker AW, Roehe R, Watson M (2019). Compendium of 4,941 rumen metagenome-assembled genomes for rumen microbiome biology and enzyme discovery. Nat Biotechnol.

[19] Olm MR, Brown CT, Brooks B, Banfield JF (2017). dRep: a tool for fast and accurate genome de-replication that enables tracking of microbial genotypes and improved genome recovery from metagenomes. ISME J.

[20] Hagen LH, Brooke CG, Shaw CA, Norbeck AD, Piao H, Arntzen MØ (2020). Proteome specialization of anaerobic fungi during ruminal degradation of recalcitrant plant fiber. ISME J.

[21] Hyatt D, Chen G-L, Locascio PF, Land ML, Larimer FW, Hauser LJ (2010). Prodigal: prokaryotic gene recognition and translation initiation site identification. BMC Bioinform.

[22] Elias JE, Gygi SP (2007). Target-decoy search strategy for increased confidence in large-scale protein identifications by mass spectrometry. Nat Methods.

[23] Deusch S, Seifert J (2015). Catching the tip of the iceberg - evaluation of sample preparation protocols for metaproteomic studies of the rumen microbiota. Proteomics.

[24] Bonn F, Bartel J, Büttner K, Hecker M, Otto A, Becher D (2014). Picking vanished proteins from the void: how to collect and ship/share extremely dilute proteins in a reproducible and highly efficient manner. Anal Chem.

[25] Eng JK, Jahan TA, Hoopmann MR (2013). Comet: an open-source MS/MS sequence database search tool. Proteomics.

[26] Pedrioli PGA (2010). Trans-proteomic pipeline: a pipeline for proteomic analysis. Methods Mol Biol.

[27] Eng JK, Hoopmann MR, Jahan TA, Egertson JD, Noble WS, MacCoss MJ (2015). A deeper look into Comet-implementation and features. J Am Soc Mass Spectrom.

[28] Keller A, Nesvizhskii AI, Kolker E, Aebersold R (2002). Empirical statistical model to estimate the accuracy of peptide identifications made by MS/MS and database search. Anal Chem.

[29] Shteynberg D, Deutsch EW, Lam H, Eng JK, Sun Z, Tasman N, et al. iProphet: multi-level integrative analysis of shotgun proteomic data improves peptide and protein identification rates and error estimates. Mol Cell Proteom. 2011;10: M111.007690. 10.1074/mcp.M111.007690 PMC3237071 21876204

[30] Nesvizhskii AI, Keller A, Kolker E, Aebersold R (2003). A statistical model for identifying proteins by tandem mass spectrometry. Anal Chem.

[31] Kanehisa M (2000). KEGG: Kyoto encyclopedia of genes and genomes. Nucleic Acids Res.

[32] Buchfink B, Xie C, Huson DH (2015). Fast and sensitive protein alignment using DIAMOND. Nat Methods.

[33] Zhang H, Yohe T, Huang L, Entwistle S, Wu P, Yang Z (2018). dbCAN2: a meta server for automated carbohydrate-active enzyme annotation. Nucleic Acids Res.

[34] Konishi T, Matsukuma S, Fuji H, Nakamura D, Satou N, Okano K (2019). Principal component analysis applied directly to sequence matrix. Sci Rep.

[35] Clark PJ, Evans FC (1954). Distance to nearest neighbor as a measure of spatial relationships in populations. Ecology..

[36] Segata N, Börnigen D, Morgan XC, Huttenhower C (2013). PhyloPhlAn is a new method for improved phylogenetic and taxonomic placement of microbes. Nat Commun.

[37] Keck F, Rimet F, Bouchez A, Franc A (2016). phylosignal: an R package to measure, test, and explore the phylogenetic signal. Ecol Evol.

[38] Molina-Venegas R, Rodríguez MÁ (2017). Erratum to: revisiting phylogenetic signal; strong or negligible impacts of polytomies and branch length information?. BMC Evol Biol.

[39] Wilkinson L (2011). ggplot2: elegant graphics for data analysis by Wickham, H. Biometrics.

[40] Kanehisa M, Goto S, Sato Y, Furumichi M, Tanabe M (2012). KEGG for integration and interpretation of large-scale molecular data sets. Nucleic Acids Res.

[41] Stone L, Roberts A (1990). The checkerboard score and species distributions. Oecologia.

[42] Sasson G, Ben-Shabat SK, Seroussi E (2017). Heritable bovine rumen bacteria are phylogenetically related and correlated with the cow’s capacity to harvest energy from its feed. MBio.

[43] Shi W, Moon CD, Leahy SC, Kang D, Froula J, Kittelmann S (2014). Methane yield phenotypes linked to differential gene expression in the sheep rumen microbiome. Genome Res.

[44] Salvato F, Hettich RL, Kleiner M (2021). Five key aspects of metaproteomics as a tool to understand functional interactions in host-associated microbiomes. PLoS Pathog.

[45] Mills RH, Vázquez-Baeza Y, Zhu Q, Jiang L, Gaffney J, Humphrey G (2019). Evaluating metagenomic prediction of the metaproteome in a 4.5-year study of a patient with crohn’s disease. mSystems.

[46] Andersen TO, Kunath BJ, Hagen LH, Arntzen MØ, Pope PB (2021). Rumen metaproteomics: closer to linking rumen microbial function to animal productivity traits. Methods.

[47] Snelling TJ, Wallace RJ (2017). The rumen microbial metaproteome as revealed by SDS-PAGE. BMC Microbiol.

[48] Honan MC, Greenwood SL (2020). Characterization of variations within the rumen metaproteome of Holstein dairy cattle relative to morning feed offering. Sci Rep.

[49] Rubino F, Carberry C, M Waters S, Kenny D, McCabe MS, Creevey CJ (2017). Divergent functional isoforms drive niche specialisation for nutrient acquisition and use in rumen microbiome. ISME J.

[50] Hecht N, Monteil CL, Perrière G, Vishkautzan M, Gur E (2021). Exploring protein space: from hydrolase to ligase by substitution. Mol Biol Evol.

[51] Tian L, Wang X-W, Wu A-K, Fan Y, Friedman J, Dahlin A (2020). Deciphering functional redundancy in the human microbiome. Nat Commun.

[52] Ma ZS (2020). Testing the Anna Karenina Principle in Human Microbiome-Associated Diseases. iScience.

[53] Lavrinienko A, Tukalenko E, Kesäniemi J, Kivisaari K, Masiuk S, Boratyński Z (2020). Applying the Anna Karenina principle for wild animal gut microbiota: Temporal stability of the bank vole gut microbiota in a disturbed environment. J Anim Ecol.

[54] Perez-Riverol Y, Bai J, Bandla C, García-Seisdedos D, Hewapathirana S, Kamatchinathan S (2022). The PRIDE database resources in 2022: a hub for mass spectrometry-based proteomics evidences. Nucleic Acids Res.

