# Supplementary material for: Metaproteome plasticity sheds light on the ecology of the rumen microbiome and its connection to host traits
Source: ISME J. 2022 Aug 16;16(11):2610–21. doi: 10.1038/s41396-022-01295-8 (PMC9563048; doi:10.1038/s41396-022-01295-8)
Supplement: Supplementary file 1 — Supplementary Figures and Legends [file 41396_2022_1295_MOESM1_ESM.pdf]

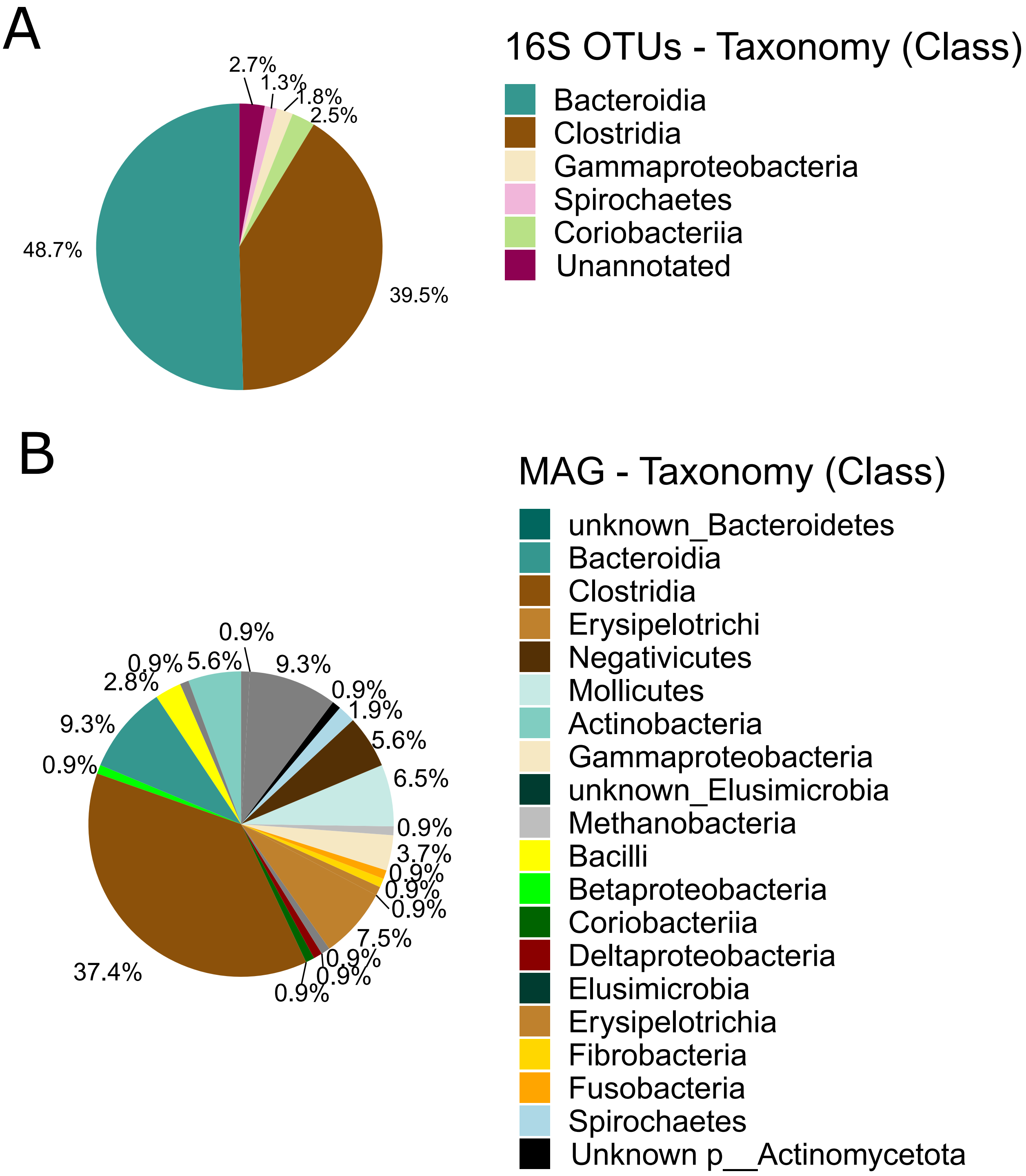

Supplementary Figure 1. Taxonomic distribution of the MAGs assembled from the rumen metagenomes. Color-coded chart pie representation of the distribution of the taxonomies at the class level of the OTUs (A) and genomic bins (B).

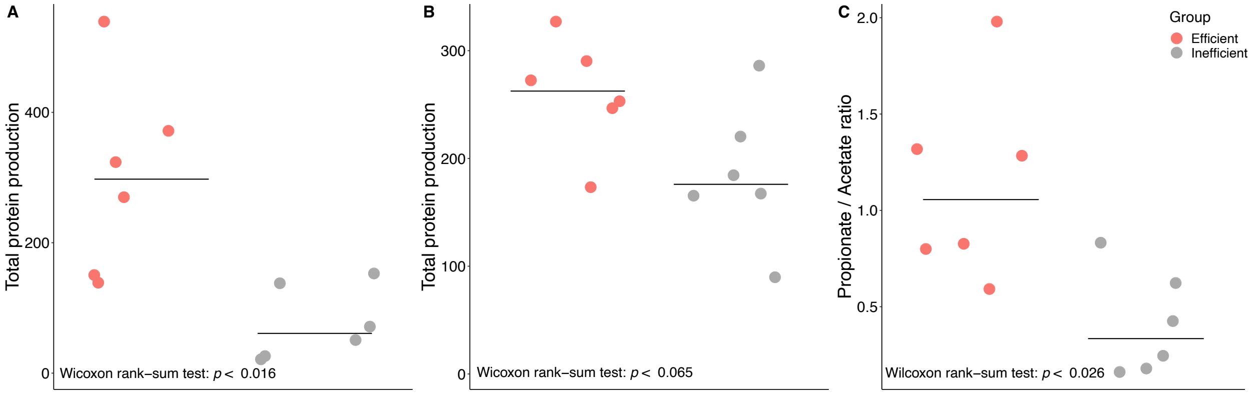

Supplementary Figure 2: Efficient animals produce higher levels of short chain fatty acid related genes, compared to inefficient animals. A. Bar plot showing the total production of propionate metabolism related proteins in efficient versus inefficient animals. B. Bar plot showing the total production of acetate metabolism related proteins in efficient versus inefficient animals. C. Bar plot showing the ratio between total production between propionate to acetate metabolism related genes, in efficient versus inefficient animals.

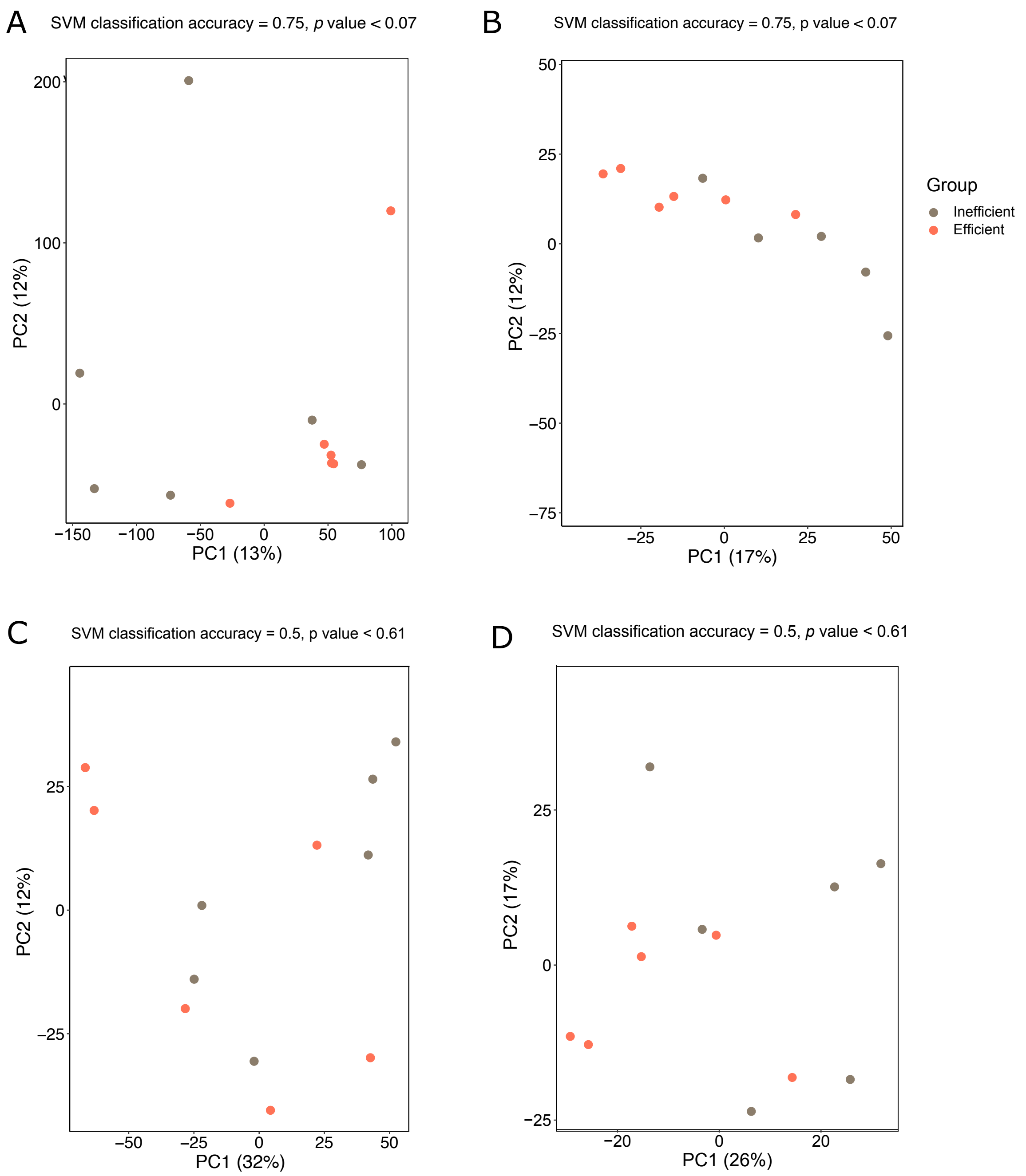

Supplementary Figure 3: Microbial abundance or raw protein production are unable to significantly differentiate feed efficient from inefficient animals in the examined samples. Ordination plots of the 12 samples using 16S rRNA amplicon sequencing (A), metagenomic abundance of the 5574 proteins that were shown to be produced in the proteomic analysis (B), non-normalized protein production of the total 5574 proteins identified in the proteomic analysis (C) and non-normalized protein production from the examined MAGS (D).

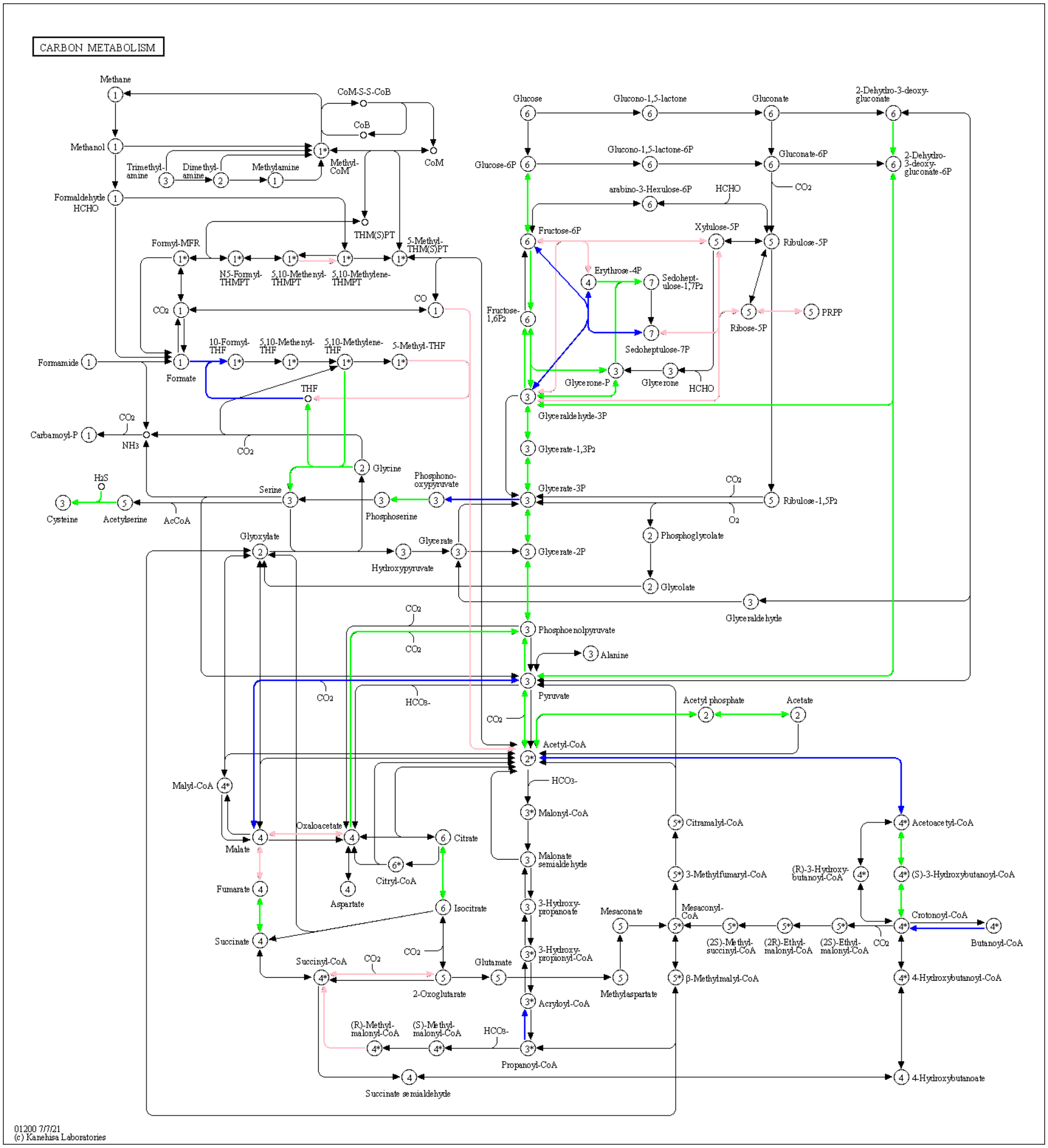

Supplementary Figure 4: KEGG map of carbon metabolism representing the identified produced KO correlated with feed efficient animals (in pink), feed inefficient animals (in blue) or both (in green) KEGG maps.

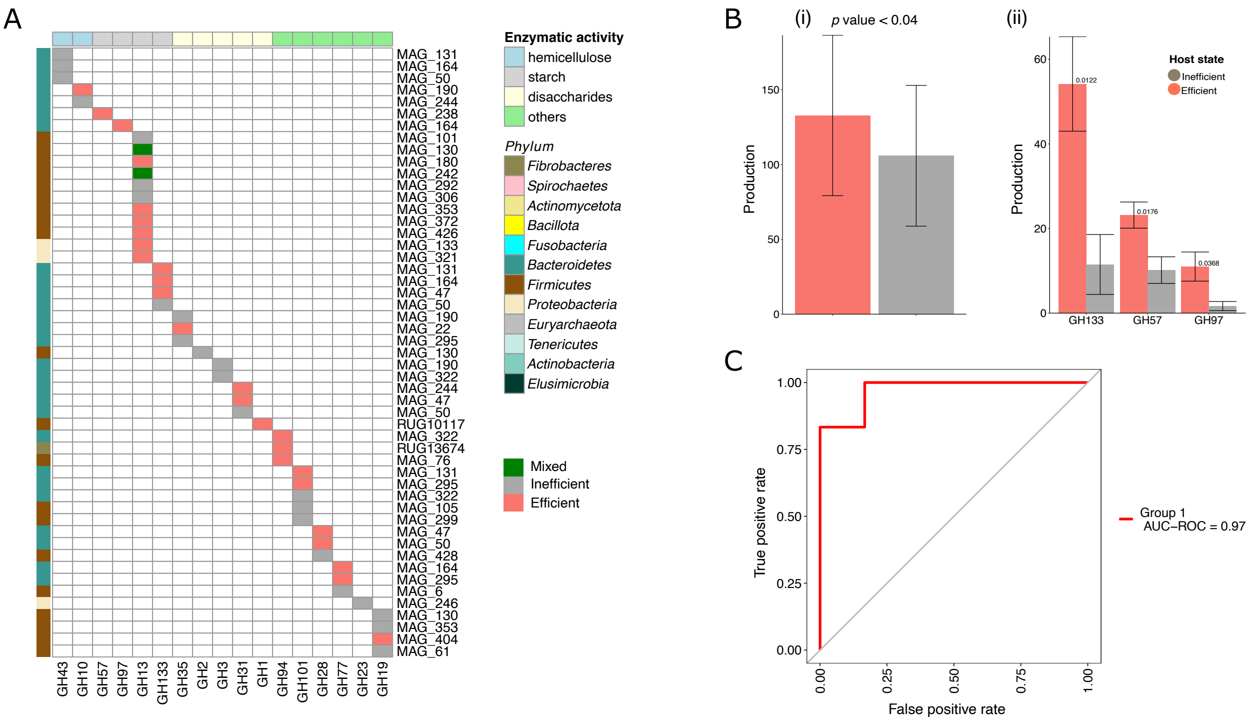

Supplemental Figure 5: Bar chart representing the actual number of shared KOs between the feed efficiency states (in gold) or the expected number of shared KOs obtained using a null model (in black). Null model was generated by randomly splitting the core proteins into random sets of the same sizes as the two actual sets of feed efficiency and inefficiency associated proteins and calculating the count of shared proteins (1,000 iterations).

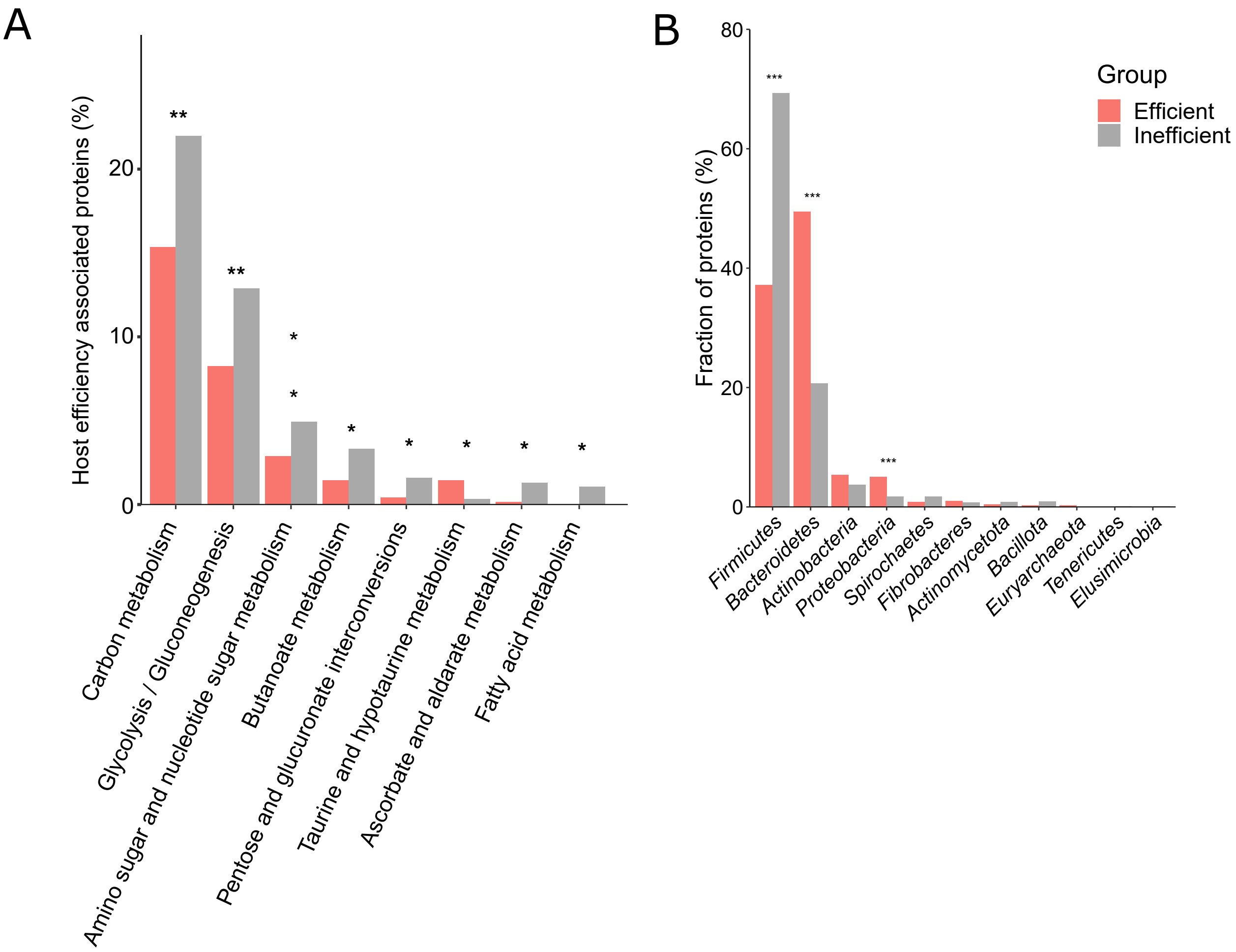

Supplementary Figure 6: Functional and taxonomic divergence of the associated proteins from the two feed efficiency groups. (A). Bar plot showing the KEGG functional category (X-axis) of the feed efficiency or inefficiency associated proteins in percentage (Y-axis). Asterisk designates significant difference, *p value* < 0.05 (Fisher Exact test). (B) Taxonomic distribution plot showing the percentage of microbial proteins (Y-axis) from a given phylum (X-axis) that are associated in the two feed efficiency states. Enrichment test was performed using absolute counts. The significant *p* value asterisks * or *** stand for $p < 0.05$ or $p < 0.0005$ respectively (Fisher Exact test).

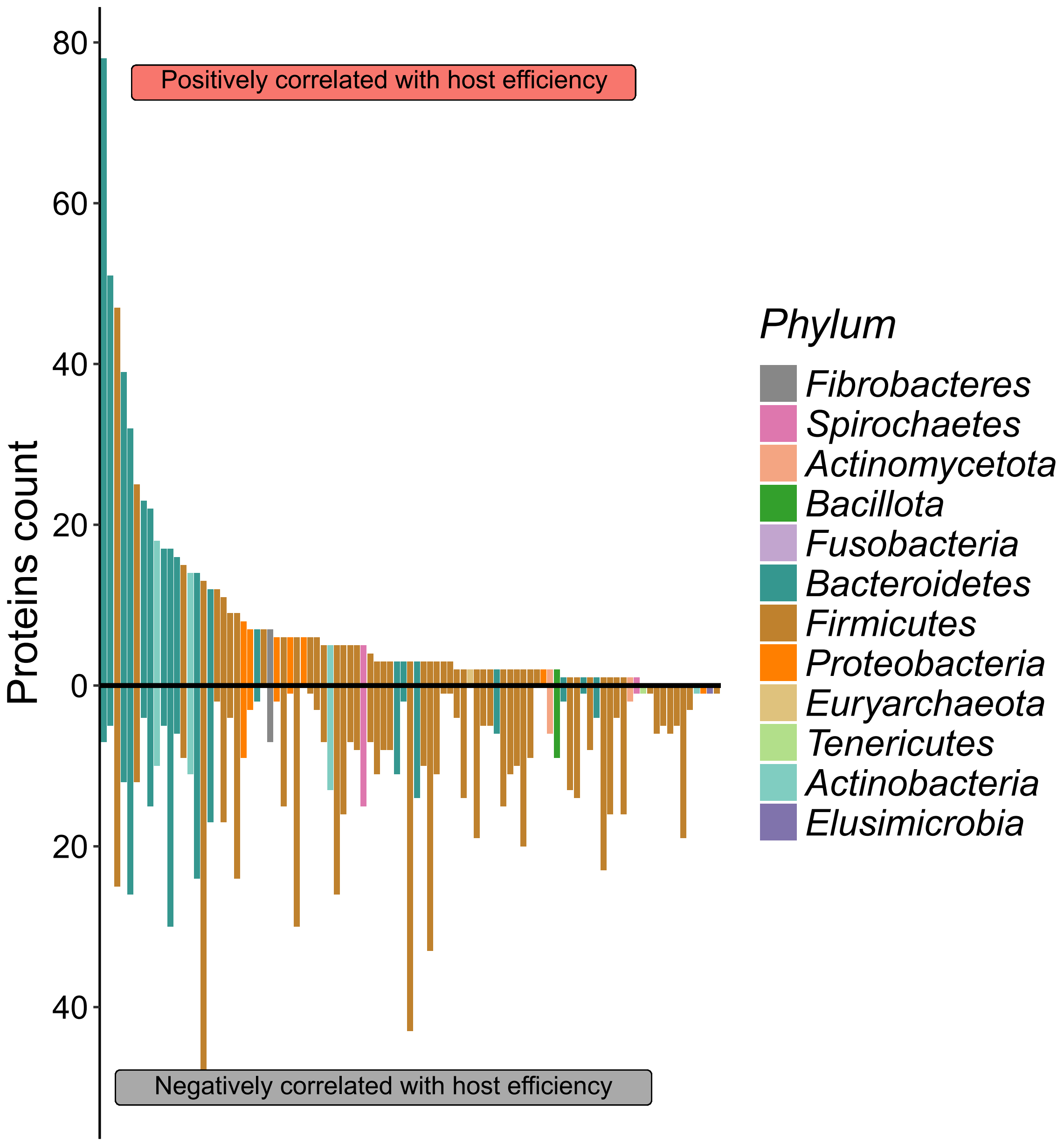

Supplementary Figure 7: Distribution of feed efficiency or inefficiency associated proteins across the different MAGs.

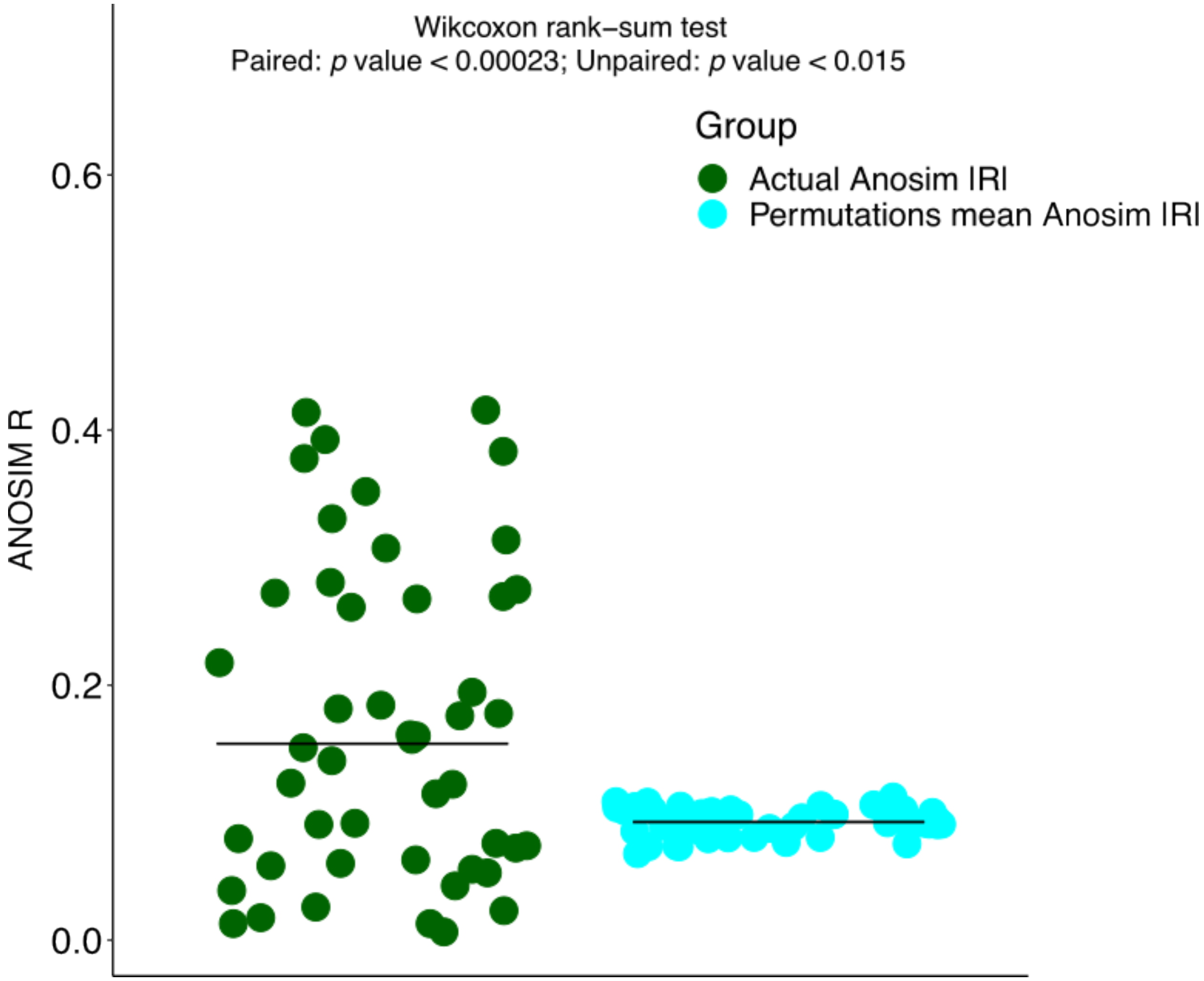

Supplementary Figure 8: Random model for MAGs differential protein production. The plot displays the comparison of the actual ANOSIM values obtained by comparing the Jaccard dissimilarity metric of the differential protein production of each MAG in either feed efficient or inefficient animals and the ANOSIM values obtained by comparing the Jaccard dissimilarity metric obtained by randomly permuting the protein production data across the different MAGs. We see that the actual values are significantly higher that the permuted values using paired Wilcoxon test $p < 0.001$.

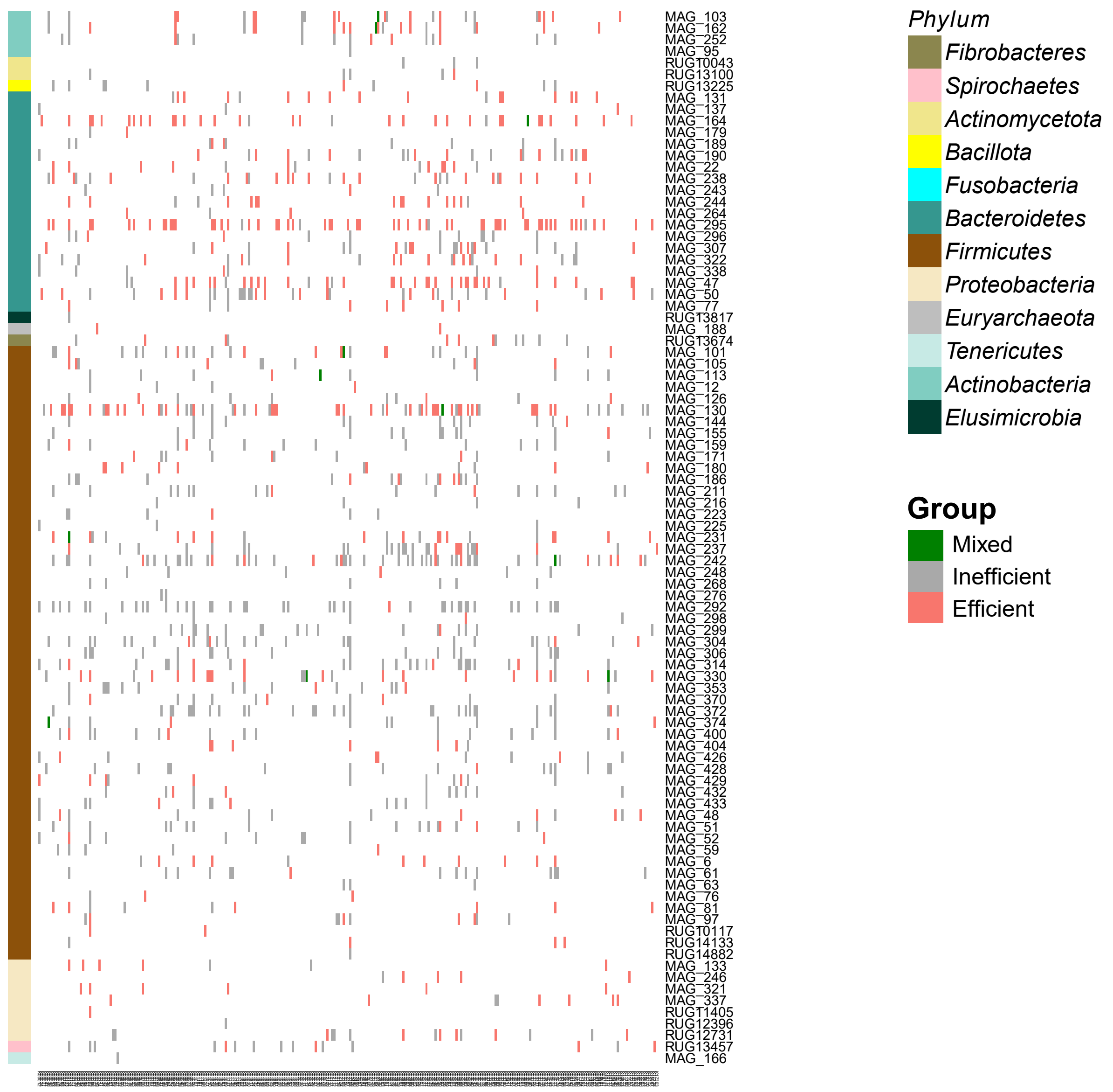

Supplementary Figure 9: Presence-absence heatmap of the produced KOs by each of the 107 MAG in feed inefficient, efficient or both host states. The rows represent a given MAG (number and phylogenies are indicated). Columns heatmap represent specific KO number grouped per MAG.

Supplementary Table legends

Supplementary Table 1: Proteomic analysis statistics. The table includes total protein counts per sample, total contaminant proteins removed per sample and average protein count per MAG per sample.

Supplementary Table 2: OTU table obtained from 16S rRNA amplicon sequencing of the rumen samples of the 12 selected animals.

Supplementary Table 3: Extended information on the 5574 proteins detected in this study, comprising protein id, KEGG BRITE functional hierarchies, KO number (if available), MAG id, MAG taxonomy and Spearman correlations, *p* values and efficiency group for core proteins.
